# Wavelength Dependence of fs Laser Ablation Ionisation Mass Spectrometry: a Dedicated Study on NIST SRM 664

**DOI:** 10.1002/rcm.70012

**Published:** 2025-12-16

**Authors:** Valentine Grimaudo, Andreas Riedo, Marek Tulej, Peter Wurz

**Affiliations:** ^1^ Physics Institute, Space Research and Planetary Sciences University of Bern Bern Switzerland

**Keywords:** femtosecond laser, IR, laser ablation, LIMS, NIST SRM 664, steel alloy, UV

## Abstract

**Rationale:**

Laser ablation mass spectrometry provides fast and direct chemical information of solids with high spatial resolution without the need for complex sample preparation. It has been shown that reducing the laser pulse length below picoseconds improves the quantification of chemical composition measurements of solids. This study compares the impact on chemical quantification of applying femtosecond laser ablation from IR to UV wavelengths to a steel alloy sample from NIST.

**Methods:**

A compact laser ablation ionisation mass spectrometer (LIMS), coupled to a fs laser ablation ion source with a fundamental laser wavelength of 775 nm, is used for the presented mass spectrometric results. The fundamental wavelength is frequency doubled or tripled to 387 and 258 nm, respectively. An extensive mass spectrometric campaign, comprising various laser pulse energies, was conducted using the NIST iron alloy standard reference material 664. The recorded element composition was compared with the NIST certified values.

**Results:**

Chemical composition analysis demonstrated the presence of significant chemical inhomogeneity of the NIST reference samples at spatial scales of about 10 μm, particularly in Ti and S. Large deviations identified were avoided for the presented study. A score was calculated for each of the accumulated spectra, indicating how close the measured composition reflected the certified values. The results show only minor differences between the wavelengths applied for sufficiently high irradiances.

**Conclusions:**

These studies conducted on steel alloy NIST SRM 664 demonstrate that the impact of laser wavelength on the quantification becomes only noticeable for low irradiances. At sufficiently high laser irradiances at around 3.1 TW/cm^2^ and beyond, comparable calibration coefficients can be observed.

## 1 Introduction

Laser ablation mass spectrometric (i.e., laser ablation inductively coupled plasma mass spectrometry (LA‐ICP‐MS [1, 2]) and laser ablation ionisation mass spectrometry (LIMS) [3, 4]) and spectroscopic systems (i.e., laser‐induced breakdown spectroscopy (LIBS) [5, 6]) are powerful tools for the chemical analysis of complex solids that are of interest to various fields of academic and industrial research. Such systems are used in a wide range of applications, ranging from the chemical analysis in geology and planetary sciences to the detailed investigation of layered systems [7, 8, 9, 10, 11, 12, 13]. The laser ablation ionisation ion source can be arranged to high lateral resolution at the micrometre level and allow material ablation with a vertical resolution down to the nanometre level [14, 15, 16]. Among the aforementioned techniques, LA‐ICP‐MS is the most widespread tool in both industry and academia. Each of these systems has its advantages and drawbacks in terms of detection sensitivity, accuracy, applicability to different samples, dynamic range, measurement time, etc. The negligible sample preparation required before analysis, however, makes all of them attractive for analytical applications, especially if limited sample material is available.

In remotely controlled instruments, such as in situ chemical analysis of geological materials or production lines, direct measurement strategies that do not require the application of standard reference materials (SRMs) for quantification are of significant interest. This will allow a decrease in the measurement time and avoid complexity with the selection of appropriate matrix‐matched reference materials. Moreover, often appropriate matrix‐matched reference samples that are required for accurate laser ablation‐based mass spectrometry are limited and only close‐matched standards can be used, limiting somewhat the accuracy of the determination of calibration factors [17, 18]. A big step forward was achieved by introducing laser systems with pulse widths shorter than picoseconds [19, 20, 21]. The application of femtosecond laser systems shows improved efficiency in the ablation of any material (glass, semiconductor, metal) and avoids localised thermal alteration of the sample material, such as sample damage, melting or recrystallisation, which are known to promote severe elemental fractionation during chemical analysis while applying laser systems providing longer laser pulses [22]. The effect of the pulse length on material ablation was intensively studied experimentally and theoretically so far due to its fundamental interest and extended applications in material sciences [23, 24]. Typically, for laser pulse durations longer than ps, various effects resulting from the laser‐plasma interaction can occur and affect plasma temperature and plasma chemistry, thus affecting ionisation processes and ultimately the quantitative composition analysis.

Even though improved performance is observed by applying fs laser systems, the laser–matter interaction is complex and could be different for different materials, including metals, semiconductors and dielectrics. fs laser pulses are sufficiently short that the plasma is produced right at the surface and evolves freely from the surface; hence, laser–plasma interaction is avoided. It has been shown that fs laser ablation sources improve the quantitative performance of both LA‐ICP‐MS and LIMS methods compared with longer pulsed laser systems [3, 25]. In LA‐ICP‐MS, one of the reasons is the production of favourable small‐size particles (nanometre‐size particles) during material ablation. In LIMS, typically one observes increased intensities of ions, which are typically difficult to ionise due to their first high IP. fs laser pulse duration allows achieving high laser intensities, which is of considerable importance for high energy gap materials such as semiconductors or dielectric materials, where laser intensity has to be sufficiently high to allow deposition of laser energy through photo ionisation (free electron generation in the conduction band). Considering that the energy transfer time from the electrons to ions by Coulomb interactions is significantly longer compared with the fs‐laser pulse duration, other forces become significant for momentum transfer from the laser field and energetic electrons and have their origin in the electric field charge separation and in ponderomotive force [26, 27]. For fs laser irradiance above the ablation threshold, the atomic ions are formed at the surface at times shorter than fs laser pulse duration and form conditions for further laser photon absorption by free electrons due to inverse Bremsstrahlung and resonance multiphoton absorption [28]. For the charge separation, the energy absorbed by electrons exceeding the Fermi energy (sum of binding energy and work function) is required. Thus, the electrons can escape from the surface and pull the ions out via Coulombic explosion. In contrast, the ponderomotive force pushes electrons formed at the surface deeper into the material structure. Therefore, the ionisation process is highly nonlinear, and its performance can be further increased by careful control of the applied laser irradiance (pulse energy (PE) divided by spot area and pulse width). The irradiance is especially important for LIMS systems, as they measure directly the ions generated during the laser ablation and ionisation. Through pulse shape optimisation, the quantification can be further increased. For further information about all these parameters, we refer to a recent review from Tulej et al. [3].

The significant improvements in the relative sensitivity coefficients (RSCs), which are derived from SRMs and used as calibration factors to calculate the effective element abundance of a solid (matrix matched with SRM), were reported in several studies [19, 29, 30]. So far, most of the fs laser ablation studies have been conducted with IR fs pulses. With the availability of simple and robust harmonic generators providing shorter wavelengths, the fs pulses with sufficiently high pulse energies down to UV can be generated and applied for material ablation. A few studies with fs laser pulses of shorter wavelengths have indicated so far an improved ablation and ionisation efficiency, for example, recent profiling investigations where high depth resolution was accomplished [14]. Furthermore, using fs laser pulses for ablation results in lesser dependence of the ablation efficiency on the sample chemical composition and physical characteristics. This may also be an important factor in the quantitative performance in the chemical composition measurements and in conducting quantitative measurements without using reference sample material for the calibration.

So far, to our best knowledge, there are no systematic studies analysing the dependence of the fs pulse wavelength on the ablation ionisation efficiency and its impact on the accuracy for the quantitative chemical analysis. In this study, the element composition of NIST SRM 664 sample is measured by a LIMS system by applying a fs laser ablation ion source at three different fs laser wavelengths, 258, 387 and 775 nm. For the analysis of the quantification performance, the RSC scoring analysis is conducted for the analysis of the investigated instrument parameters (wavelength and laser irradiance).

## 2 Experimental Section

### 2.1 Sample Material

To verify the influence of the laser wavelength on the chemical composition quantification, we decided to perform all measurements on a steel alloy SRM from the National Institute of Standards and Technology (NIST): the high carbon steel sample NIST SRM 664. As a consequence, the results and implications presented in the following are restricted to metals only. SRM 664 is reported to be specifically dedicated to applications in microanalysis methods such as electron probe microanalysis, spark source MS and laser probe analysis. The investigated elements and their abundances in weight percent (wt%) are listed in Table 1.

**TABLE 1:** Certified element abundances of the high carbon steel sample SRM 664 by NIST for elements lighter than Fe.

Element	NIST certified abundance (wt%)
B	0.011 ± 0.001
C	0.871 ± 0.005
Si	0.066 ± 0.001
P	0.01 ± 0.001
S	0.025 ± 0.001
Ti	0.23 ± 0.01
V	0.106 ± 0.005
Cr	0.066 ± 0.005
Mn	0.258 ± 0.005

Before chemical composition analysis using LIMS, such types of solid metallic reference materials are rinsed intensively using ethanol or isopropanol following the use of MilliQ water (MilliPore, 18.2 MΩ·cm, < 3 ppb total organic carbon), sonication within ethanol or isopropanol following MilliQ water (~15 min) and finally applying argon ion sputtering within the setup's sample port before moving the clean sample into the main vacuum chamber. After analysis, the samples are placed and stored for subsequent use within oil‐free exiccators evacuated down to few mbar levels.

Only certified elements lighter than iron were considered for analysis presented in this study to overcome possible detector‐related saturation effects caused by the intense signal of iron, which is the most abundant element in the NIST SRM 664 sample with about 99 wt% of the sample. Note that isotopes heavier than iron as well as all the iron isotopes were detected, but due to the intense peak at ^56^Fe, they are measured with a lower sensitivity. ^54^Fe was deliberately not considered because of the missing abundance specification for isotopes for that element, especially as small deviations in the major species have a severe impact on the quantification accuracy of the minor elements.

### 2.2 Laser Ablation Ionisation Mass Spectrometry

The LIMS instrument applied in this study consists of a pulsed laser ablation and ionisation source combined with a miniature mass spectrometer (MS) (Ø 60 × 160 mm). The MS is a reflectron‐type time‐of‐flight (R‐TOF) mass analyser (MA) that was conceptually designed for in situ space application on a rover or lander mission for the chemical analysis of solids, explaining its compact dimensions. The MA is currently made flight ready for a commercial lunar payload service (CLPS) mission of NASA that shall be launched in 2029 [31, 32, 33]. In the present study, we used a chirped pulse amplification (CPA) Ti:Sapphire laser, with a fundamental output wavelength of *λ* = 775 nm, *τ* ~ 190 fs, maximum PE of up to about 1 mJ and providing a laser pulse repetition rate of up to 1 kHz. The fundamental beam is directed via dielectric mirrors towards a harmonic generator unit (Storc, Clark‐MXR Inc.), which converts the fundamental wavelength to either 387 or 258 nm. Note that all data from the 775 nm laser light were recorded before the installation of the harmonic generator unit. The output beam is subsequently guided through a beam expander (Eksma Optics, Vilnius, Lithuania) that enlarges the laser beam to about 30 mm in diameter to achieve sharper focusing at the sample surface. The expanded beam is then guided inside the vacuum chamber and is directed to a doublet lens system with a focal length of 250 mm, which focuses the beam through the central axis of the MA towards the sample surface. The sample is located directly below the MA, about 0.5 mm away from the entrance electrode of the MA.

A remotely controlled translation stage with micrometre resolution is used to position the sample in *x*, *y* and *z* directions. Each laser pulse ablates a distinct layer of material normal to the propagation axis of the laser beam. The electrical fields at the entrance of the MA extract, confine and accelerate only positively charged ions. After having passed the field‐free region and the ion optics at the reflectron, which turns the ion trajectory in the opposite direction, parallel to the propagation path of the laser light, the ions impinge at the centrosymmetric microchannel plate detector (MCPs) in chevron configuration [34]. The integrated anode system of the detector consists of four anode rings [34] allowing signal acquisition at different gain levels [35] at the read‐out electronics consisting of two high‐speed ADC cards (Acqiris SA, Geneva, Switzerland) with a sampling rate of up to 3.2 GS/s. Further information about the LIMS system and the measurement capabilities are provided in the indicated publications [15, 36, 37, 38].

### 2.3 Measurement Procedure

In the current study, only one detector channel (1 V, 9‐bit effective resolution) was used for signal acquisition. Every measurement consisted of 100 consecutive laser bursts, where each burst comprised 1000 laser pulses. The software on the read‐out‐electronics histogrammed directly the 1000 single mass spectra from each laser burst onboard and saved it as one mass spectrum on the host computer, resulting in 100 consecutive recorded spectra per measurement run. Each measurement run was performed with a different PE. The applied PE was recorded for each complete measurement run with a J‐10 MB‐LE sensor (Coherent, Santa Clara, CA, United States) in front of the beam expander and was corrected for transmission loss (from beam expander to sample surface). The base pressure in the vacuum chamber was in the range ~10^−7^ mbar. The potential difference across the MCP stack was set to ~1,550 V.

For the wavelengths 775, 387 and 258 nm, mass spectrometric signals from 15 different PEs (2.63, 2.38, 2.16, 1.99, 1.69, 1.52, 1.27, 1.15, 1.0, 0.84, 0.73, 0.63, 0.56, 0.41 and 0.29 μJ), 10 different PEs (0.60, 0.48, 0.40, 0.31, 0.24, 0.20, 0.17, 0.13, 0.11 and 0.06 μJ) and 10 PEs (1.21, 0.95, 0.76, 0.60, 0.46, 0.36, 0.30, 0.23, 0.18 and 0.08 μJ) were recorded. For the 387 nm wavelength, five repetitions for each PE were conducted. Each measurement was conducted on a fresh sample location, with a pitch of 50 μm for the studies concerning 387 nm and 100 μm for the 775 and 258 nm campaigns.

In‐house written software packages were used for the automation of the entire instrument (laser system, data acquisition and sample stage) as well as the data processing following the measurements [39].

## 3 Results and Discussion

**FIGURE 1:**
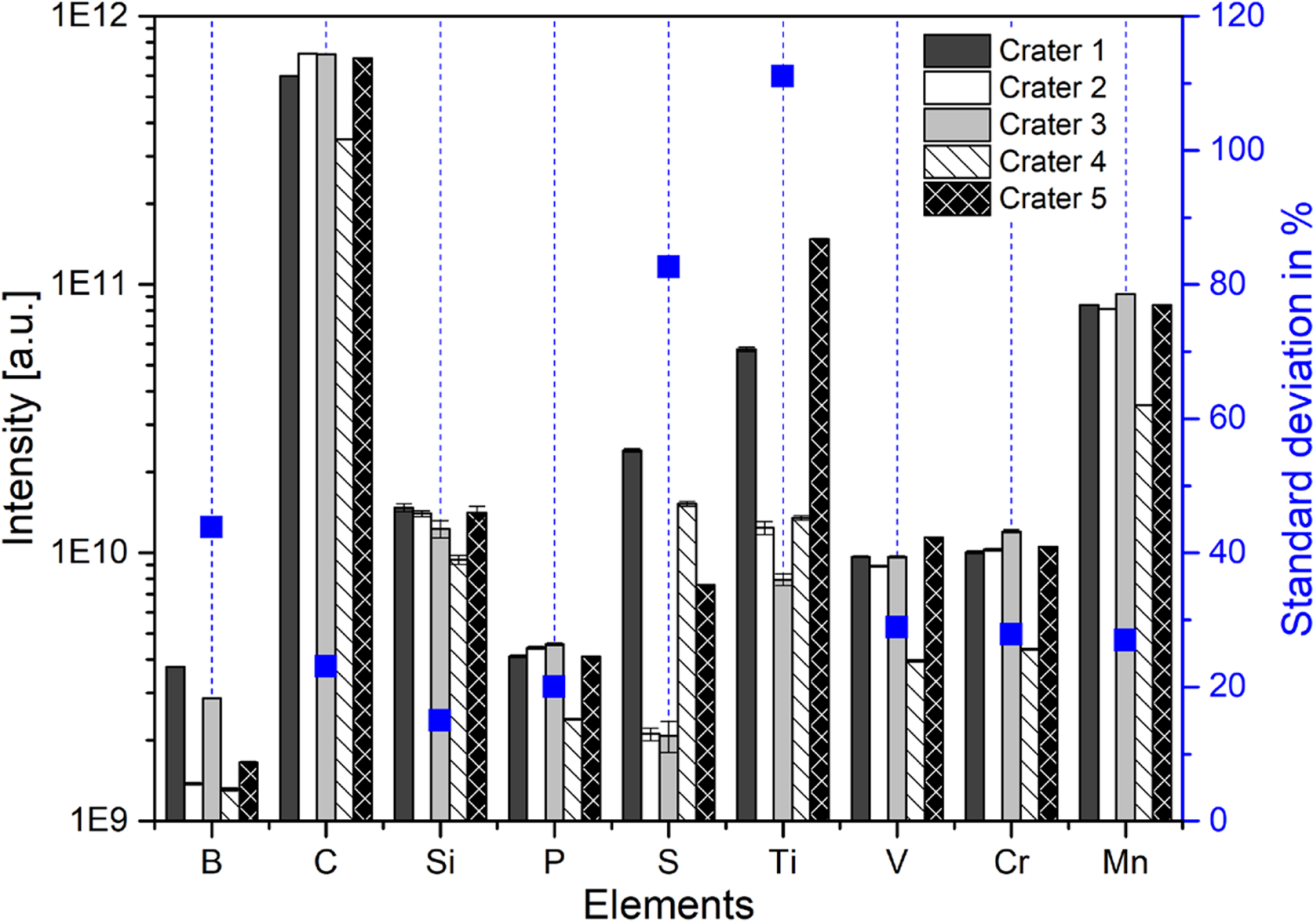
Bar plot for comparison of the measured element intensity from five different positions. The measurements were done with the laser wavelength 387 nm at a PE of 0.24 μJ. For each position, between 6200 and 9200 spectra were evaluated. The blue data points present the corresponding standard deviation, showing S and Ti being particularly nonuniformly distributed along the sample.

**FIGURE 2:**
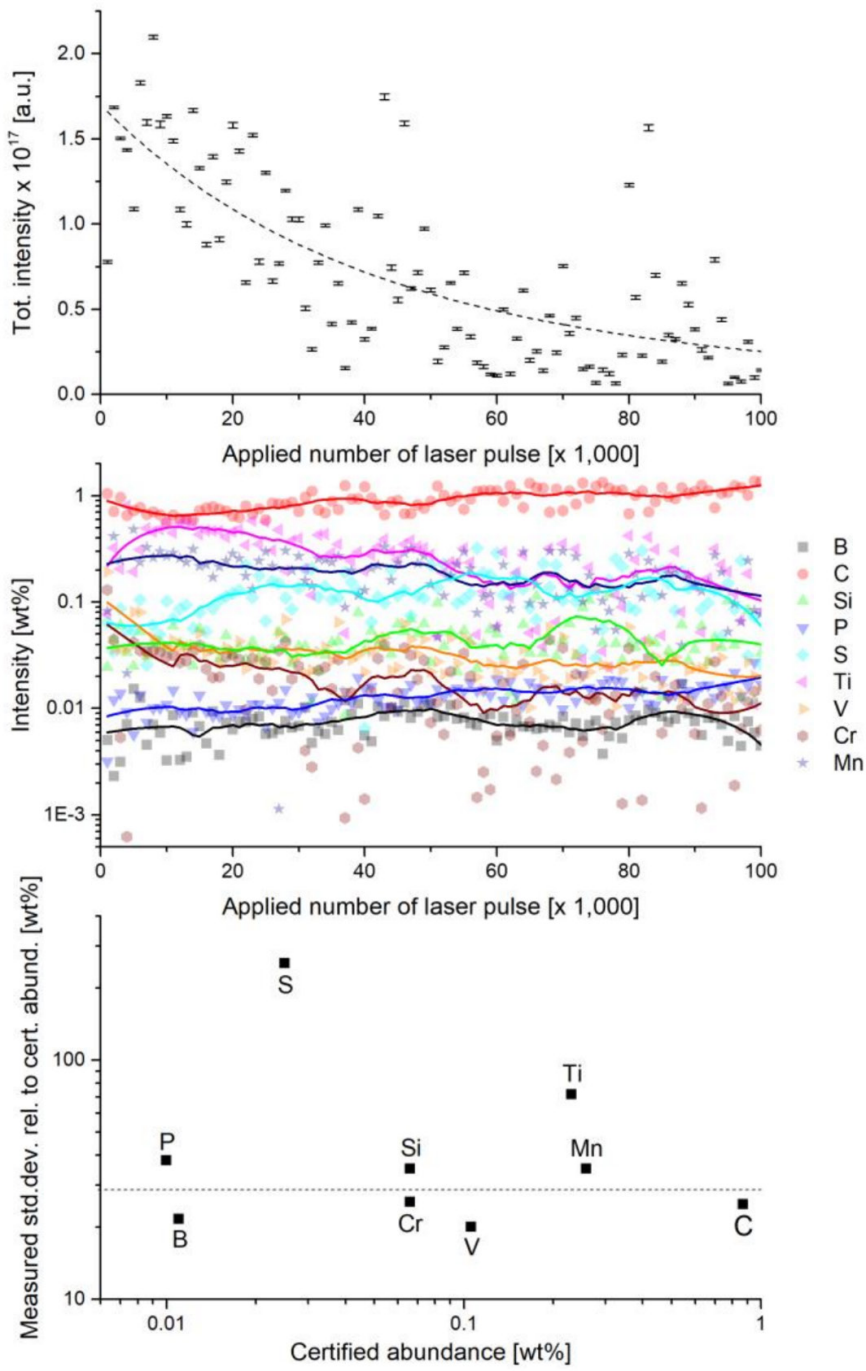
Chemical inhomogeneity along the sample depth measured with a 387‐nm laser wavelength at a PE of 0.23 μJ. Top: Exponential decrease of the sum of all considered certified element intensities (B to Mn) with successive laser pulses. Middle: Depth profile of the measured element intensities with depth, that is, increasing number of laser pulses. Element abundance variations with increasing depth can be clearly observed. Bottom: Measured standard deviation relative to the certified abundances. Most of the elements show variations in the order of about 20%–30%, except for S and Ti, which have a much stronger variation also for this measurement location. Note that the line is not a linear fit through the data; it is only meant to guide the eye.

For the comparison of the accuracy of abundance quantification between the individual measurement runs and applied wavelengths, it is mandatory to probe areas on the sample with preferably similar element composition. During the measurement campaign, however, a significant variation in chemical composition within the analysed area was observed (see Figures 1 and 2). In Figure 1, the element intensity measured with *λ* = 387 nm for the elements B to Mn from five different sample locations with a pitch distance of 50–100 μm is plotted. The intensities are derived from a histogrammed mass spectra containing several thousand single laser mass spectra. The right *y*‐axis displays the standard deviation in % from these five measurements. We observe that Ti and S have a particularly strong spot‐to‐spot abundance variation.

Chemical inhomogeneity is not only observed between the sampled locations at a spatial scale of tens of micrometres but also within a single ablation crater, as illustrated in Figure 2. The top panel shows a successive decrease in the overall intensity (sum of the peak intensities of all considered elements) with an increasing number of laser shots, as is typical for laser ablation at such spatial scales, which results from the decreasing ablation rate with increasing depth [40]. The chemical depth profiles of the relevant elements are presented in the middle panel of Figure 2. The element abundance variation observed evolves from the chemical inhomogeneity of the material that is also manifested in the depth. A measure to indicate how severe the variation can be for the individual elements is given by the standard deviation of their abundance (see Figure 1).

When plotting the standard deviation in relation to the expected element abundance, one can see that the majority of the elements show a deviation in the order of about 20% to 30% (see Figure 2, bottom panel). Note that the given line is just to guide the eye; it is not a linear fit. However, in this particular measurement location, S and Ti show a stronger variability with depth than the other elements, which is in line with the measurement done at different locations.

**FIGURE 3:**
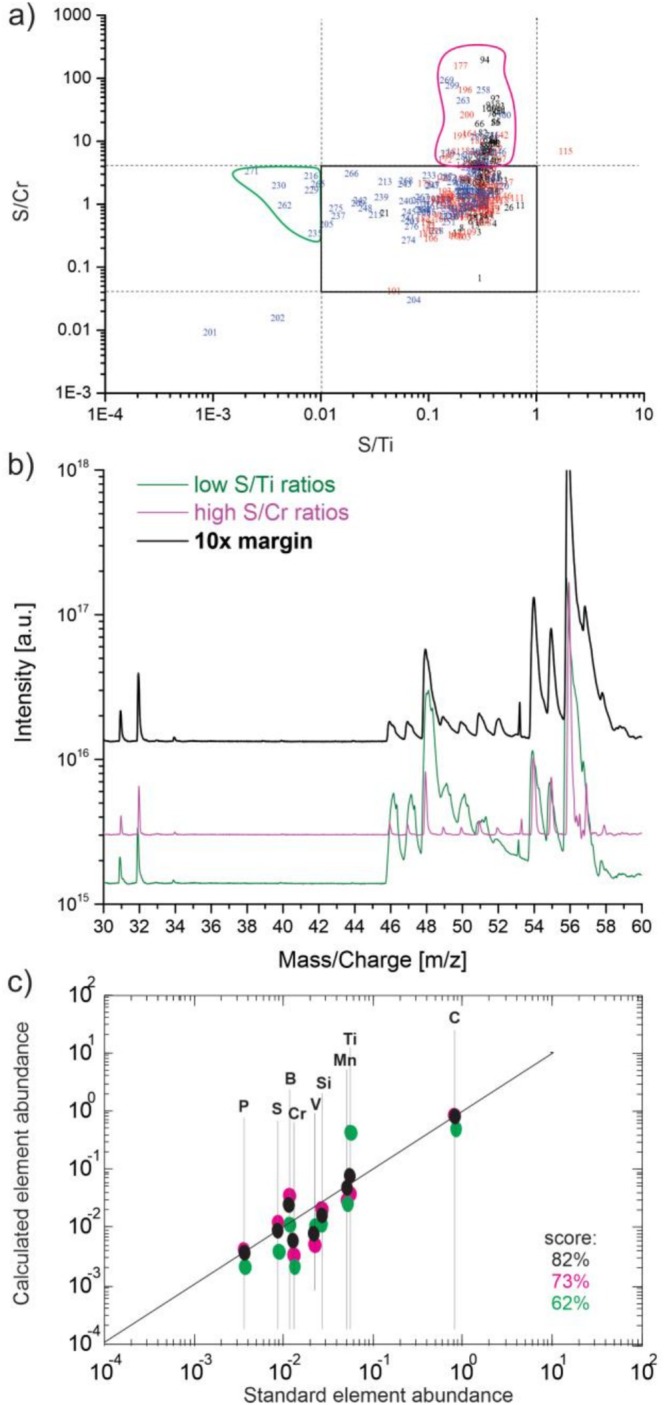
(a) Measured S/Cr and S/Ti ratios for three different sample locations (indicated in black, red and blue). Each measurement point is an accumulation of 1000 mass spectra recorded with 387 nm and 0.4 μJ/pulse. (b) Histogrammed mass spectra from the blue crater from the different chemical identities (green, pink and black square) highlighted in panel (a). (c) Calculated element abundances in correlation to the certified NIST abundances for the elements B to Mn from the mass spectra in (b). The derived RSC score of the individual accumulated mass spectra is given on the lower right corner.

The observations of abundance variability can be explained by the spatial dimension of the probed sample location, which might be smaller than the actual dimension of the involved inhomogeneity. The removed material per laser pulse comprises an area of < 10 μm in diameter and a depth of only tens of nm. To overcome the problem of abundance inhomogeneity, preprocessing of the data is required, which was carried out, where measurements with strong element composition variation were excluded from the analysis. Additionally, not well‐resolved mass spectra were rejected from the data analysis as well. Space charge effects, such as surface charging or Coulombic repulsion, affect the ion trajectories at the ablation site. Above a certain severity of these effects, they degrade the mass resolution of a mass spectrum. The analysis of a not sufficiently well resolved spectrum yields less accurate element intensity determination for some intense and congested mass peaks. These spectra can then result in misleading element ratios and are therefore not considered for subsequent analysis. To identify outliers of chemical homogeneity, a 2D correlation analysis of S/Ti and S/Cr element ratios was established. This analysis is shown in Figure 3a. Since Ti and S show the largest variability between the individual measurements, they were chosen for this analysis. Cr was selected as a reference to consider in the process of elimination because there is little abundance variation (Figure 2, bottom panel). From chemistry, it is known that Ti exhibits a high affinity for S, reacting to form Ti sulphides when heated and is stable at high temperatures in the form TiS. Since the SRM samples are metallic alloys produced from a melt, they experienced high temperatures and TiS might have formed and segregated to small TiS inclusions, changing the chemical composition locally.

The 2D correlation plot involves the measurement of three locations (red, blue and black symbols), each one probed by 100 × 1000 laser pulses, resulting in 100,000 recorded mass spectra per position in total. The plot shows a wide range of ratios in all three measurement locations. The black square in the centre of the plot encloses all measurements with S/Ti and S/Cr ratios that deviate not more than 10 times the expected value from NIST. The measurements outside that regime show either a ratio 10 times smaller (e.g., measurement points surrounded in green) or 10 times higher than the expected value (e.g., measurement points surrounded in pink). Figure 3b illustrates the difference in composition between the individual regions (black and green) of panel (a) considering mass spectrometric data from the blue crater (histogrammed spectra). The mass spectra presented in (b) are histogrammed spectra from the individual regions as indicated in panel (a). The comparison of the mass spectra clearly points out that the selection of eligible spectra for the analysis cannot be defined solely by the mass resolution. Figure 3c plots the calculated abundances of the considered elements B to Mn in relation to their certified value. The solid line in the plot is a linear function representing the optimal case, where the measured abundance matches the expected value. The closer to the line, the closer the calibration factor is to 1, which has to be applied to the measured value to obtain the quantitative element abundance.

A measure to quantify the calibration factor is the RSC, which is the ratio between the measured abundance and the certified abundance:

(1) $$\mathrm{RSC}=\frac{\text{measured abundance}}{\text{certified abundance}}$$

**FIGURE 4:**
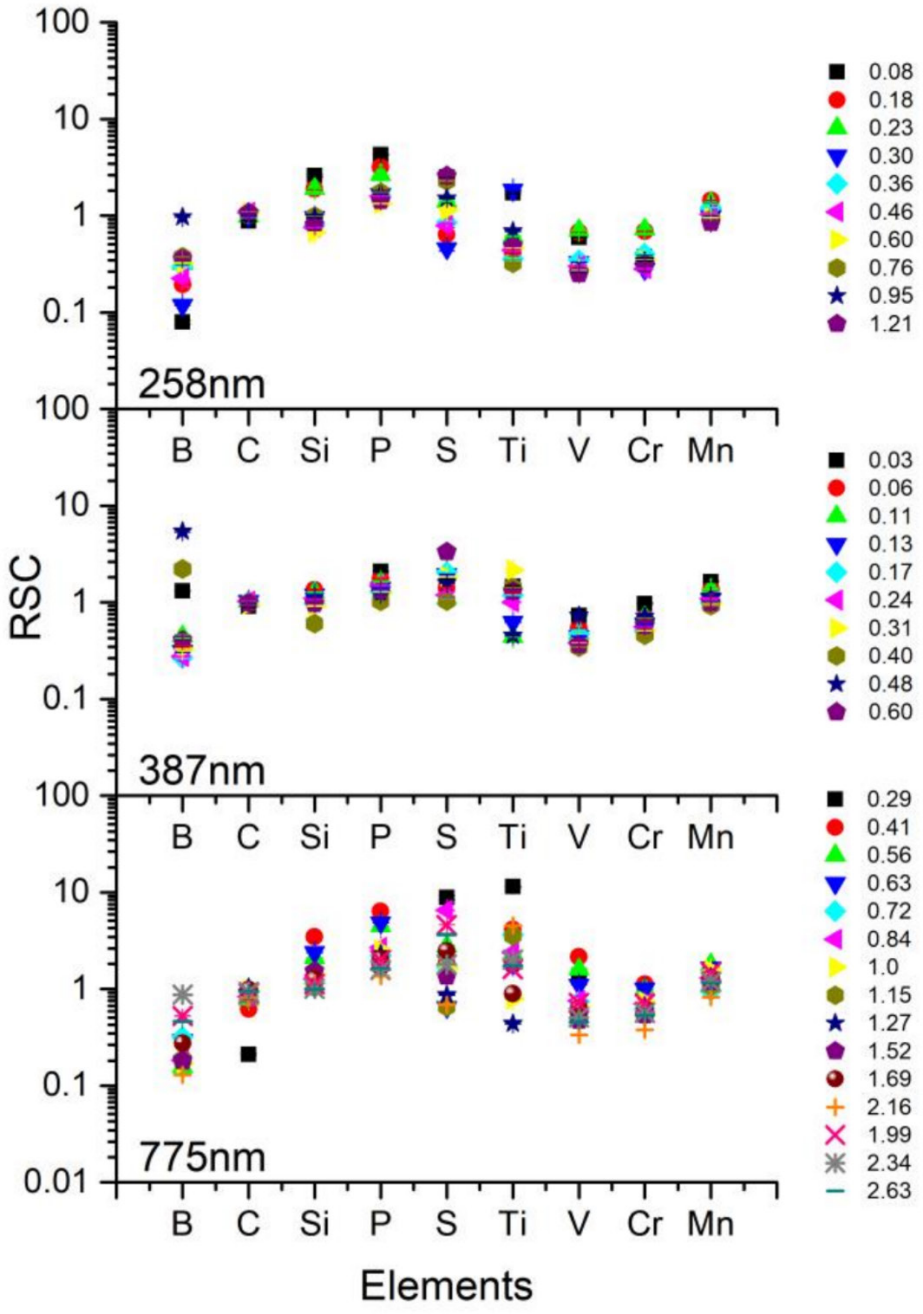
Relative sensitivity coefficient (RSC) for elements B to Mn for different laser PE (µJ) and different wavelengths (258, 387 and 775 nm). Only sufficiently resolved and mass spectra showing a S/Cr and S/Ti ratio that deviate less than 10 times the expected value were considered for the analysis.

RSC values were calculated for the elements B to Mn for a variety of PE for the three different wavelengths 258, 387 and 775 nm applied (see Figure 4), using the above‐described data pre‐processing. In this way, for example, strong localised chemical inhomogeneities are avoided and a more robust comparison with the certified values, which are bulk values, can be provided for the individual laser parameters, for example, PE and wavelengths. Table 2 lists the number of spectra that were considered for the calculation of the RSC values. A similar trend is observed over all three wavelengths; as can be seen in Figure 4, the RSC values do not show any dependence on the applied PE. The elements P and S tend to show RSC values slightly above 1, whereas B, V and Cr tend to have RSC values slightly below 1. In this representation, one can also recognise that B has a larger distribution of RSC values, like S and Ti, than the other elements, a factor that was not considered for the selection of the proper mass spectra (as comparison, Figure 3).

**TABLE 2:** Number of mass spectra considered for the calculation of the RSC values for the range of PE.

258 nm		387 nm		775 nm	
PE (μJ)	Nb. spectra	PE (μJ)	Nb. spectra	PE (μJ)	Nb. spectra
0.08	72 × 1000	0.06	80 × 999	0.29	15 × 1000
0.18	57 × 1000	0.11	71 × 999	0.41	76 × 1000
0.23	40 × 1000	0.13	83 × 999	0.56	74 × 1000
0.30	59 × 1000	0.17	90 × 999	0.63	61 × 1000
0.36	47 × 1000	0.20	94 × 999	0.73	57 × 1000
0.46	54 × 1000	0.24	76 × 999	0.84	76 × 1000
0.60	55 × 1000	0.31	63 × 999	1.00	75 × 1000
0.76	44 × 1000	0.40	66 × 999	1.15	69 × 1000
0.95	65 × 1000	0.48	86 × 999	1.27	84 × 1000
1.21	16 × 1000	0.60	70 × 999	1.52	60 × 1000
				1.69	91 × 1000
				1.99	51 × 1000
				2.16	19 × 1000
				2.38	74 × 1000
				2.63	40 × 1000

**FIGURE 5:**
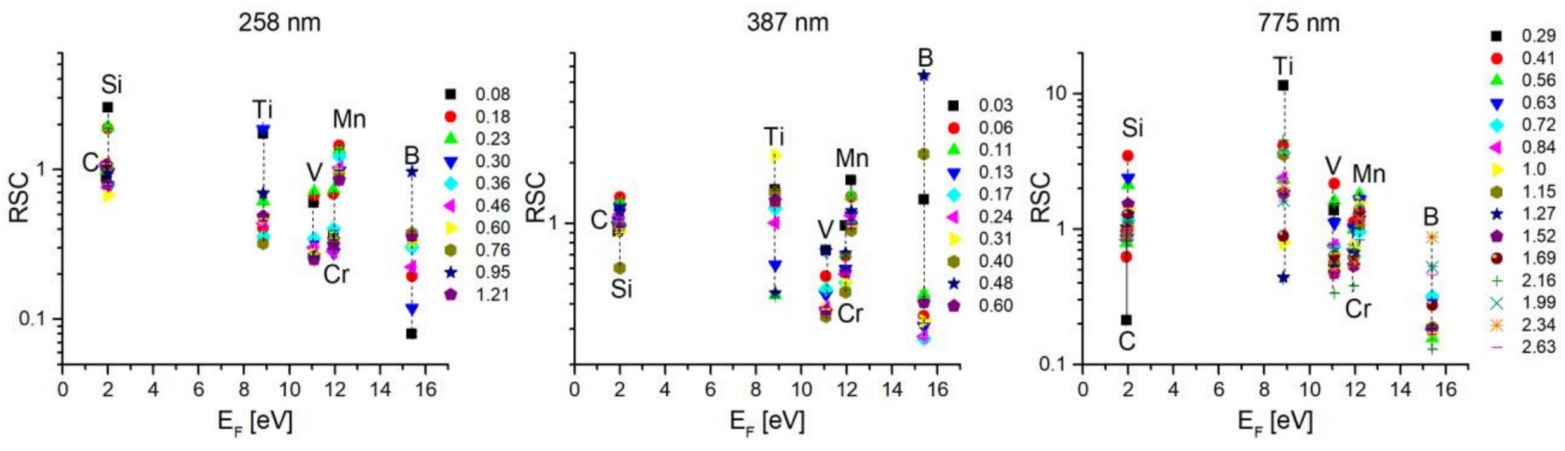
RSC dependence on fermi energy *E*_*F*_ for different PE (µJ) and wavelengths. Only sufficiently resolved mass spectra showing a S/Cr and S/Ti ratio that deviates less than 10 times the expected value were considered for this analysis.

However, when plotting the RSC values as a function of the fermi energies, which is the minimum energy required to eject an electron from the solid to the vacuum, a trend of the RSC towards smaller values for increased fermi energies *E*_*F*_ is observed (see Figure 5). Note that, because of the small laser irradiance applied with this LIMS instrument, the ionisation is expected to occur mostly right on the surface, after excitation of the solid with the fs pulse [3]. Note that the mechanism of atomic ion production at the surface was not fully explored in this study here. Nevertheless, the laser pulse energies applied in this study here for the ablation are less than a factor of about 3 to that of the observed laser ablation threshold energy. At these laser pulse conditions Coulombic explosion mechanism is mainly responsible for the ablation and ion production rather than thermal ablation mechanisms. The atomic ions are likely formed via multiphoton absorption with perhaps optical field ionisation mechanism. The ionisation efficiency is readily increasing while applying shorter wavelength laser pulses and lower irradiances compared with the results obtained for the IR laser ablation. Because in this study the accumulated mass spectra emerge from a couple of picograms of material, involving therefore also the bulk, the RSCs are expected to correlate with *E*_*F*_ only moderately. Additionally, the sample is a mixture of different elements and because the probed area with the laser is mostly larger than the size of the individual chemical components, the fermi energy of the surface is expected to be rather a convolution of individual ones.

To assess which laser wavelength is providing the best quantitative results for LIMS measurements, the RSC score was investigated for every PE. The RSC score, as specified in Equation 2, describes how close to the optimum of RSC = 1 the overall element abundance measurement was.

(2) $$\begin{array}{c} \left(1-\left[\frac{1}{9}*\left(\sum_{i=1}^{i=9}\frac{|1-{\mathrm{RSC}}_{i}|}{\left(1+{\mathrm{RSC}}_{i}\right)}\right)\right]\right)*100\% \end{array}$$

Note that *i* = 9 is mentioned in Equation (2) because nine different NIST certified elements are considered in this study, from B to Mn. The ideal measurement would provide every element with the exact certified abundance, without the application of any calibration factor, which would imply that all RSC values for all elements would be 1.

**FIGURE 6:**
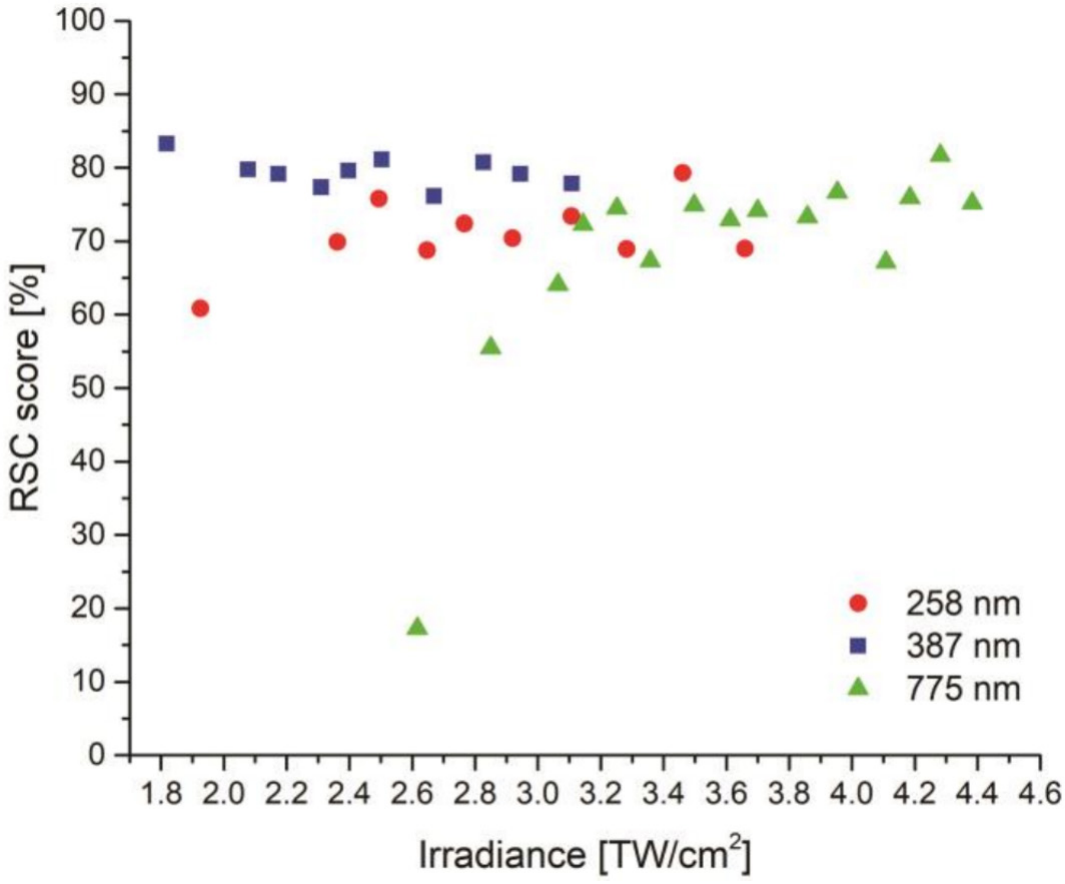
RSC score in % (Equation 2) for all three wavelengths describing how close the overall measured abundance is with respect to the optimum, in relation to the applied laser irradiance. The RSC score includes all certified elements from B to Mn. The irradiance is derived using the allometric fit function indicated in Figure S2, assuming the same crater evolution behaviour for steel SRM and the Cu‐foil sample. Further information in SI.

In Figure 6, the impact of the laser wavelength and laser PE on the quantification capability of this LIMS instrument is illustrated. For every tested PE, the corresponding RSC score was investigated. To enable the comparison of the PE, latter were converted to irradiances, which are laser power normalised to the irradiated surface. The sampled area depends on the cross‐section of the spatial profile of the laser pulse (typically Gaussian) that strikes the sample surface during the experiment, which in turn depends on the applied PE. Further information about sampled area (laser spot size on the sample, Figure S1) and the conversion from PEs to laser irradiances (Figure S2) can be found in the Supporting Information section. The correlation of the RSC score and the applied laser irradiance shows that a similar quantification capability of our LIMS instrument is achieved for different wavelengths when applying a sufficient high laser irradiance of about 3.1 TW/cm^2^ and higher; 775 nm: mean RSC score = (71.8 ± 6.5) % (excluding the first point), 387 nm: mean RSC score = (79.4 ± 2.0) % and 258 nm: mean RSC score = (72.0 ± 3.6) % (excluding the first point). With the 387 nm, an improvement of about 9% in comparison with the 775 nm was observed. Use of the third harmonic wavelength resulted in a comparable RSC score to the fundamental wavelength of 775 nm. In other words, the wavelength has only marginal impact on the accuracy of the measurement abundance of the metallic NIST SRM 664 sample when using the fs laser system at the appropriate laser irradiance for this LIMS setup. Applying IR radiation increased laser irradiances were necessary. In comparison with the shorter wavelength (387 and 258 nm), the ablation threshold was observed at higher laser irradiances, and the ion production efficiency could be increased only by the increase of the laser irradiance. Element fractionation effects, calculated as the RSC score, were observed to rapidly decrease for IR radiation towards 10% for the investigated lower laser irradiances. Such a drop is not observed for the UV radiation within the tested PE range. The lower mean RSC score for 258 nm might be explained by the lower number of considered mass spectra for detailed analysis for the RSC calculation (see Table 2). At this wavelength, a larger number of ions compared with other wavelengths is produced. As discussed earlier, space charge effects might result in not well‐resolved mass spectra, which are not considered for subsequent analysis as these spectra yield less accurate element intensity determination. Consequently, this reduces the measurement statistics and hence influences the element composition analysis of the bulk.

**FIGURE 7:**
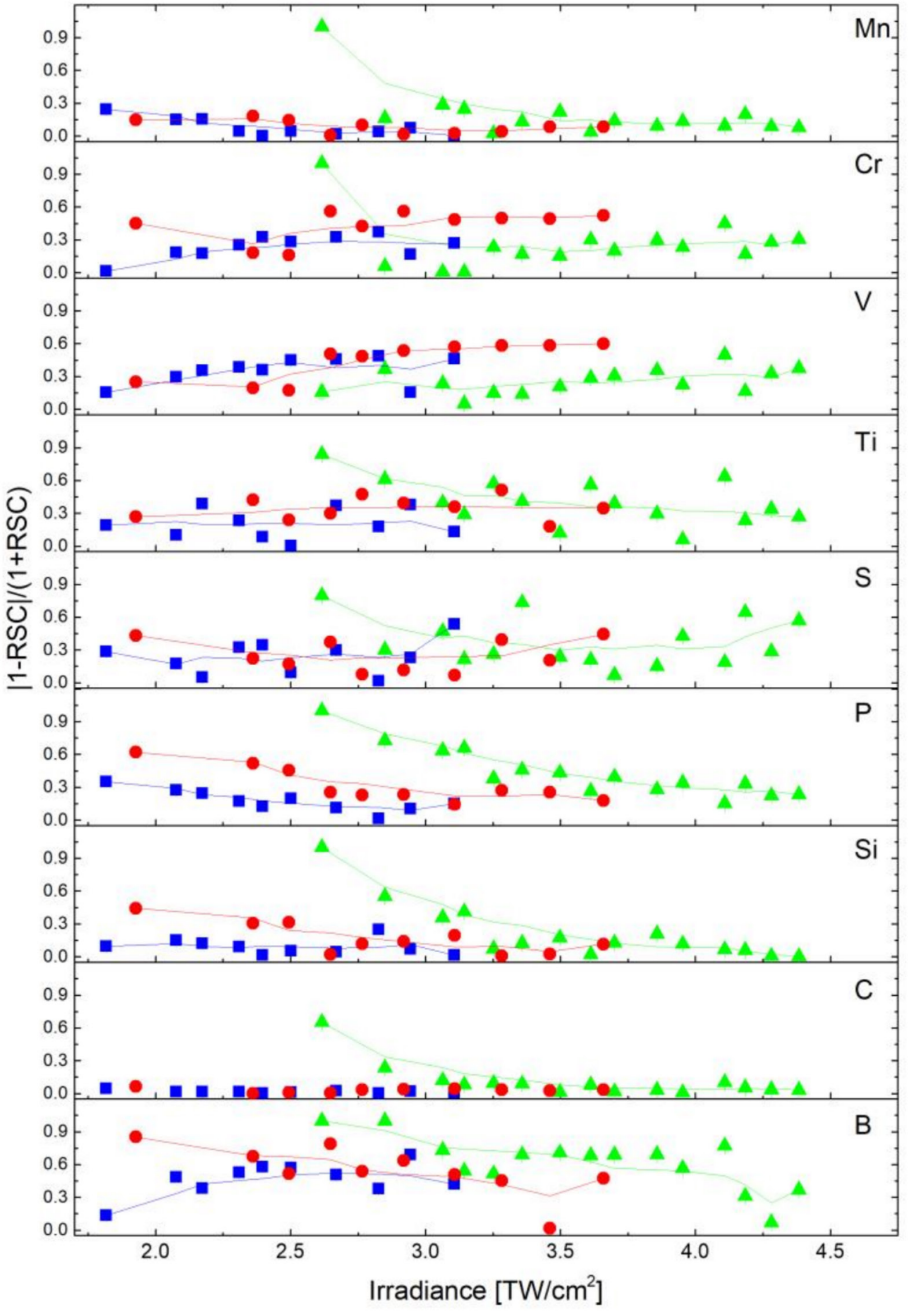
Deviation of the measured element abundance from the certified value as a function of the applied laser irradiances. The larger the value, the more deviated the measurement is. Red circles, blue squares and green triangles stand in for the data originated by the 258, 387 and 775 nm wavelengths, respectively. The line between the data points is a smoothed fit line, which averages 10 adjacent points.

In more detail, the representation in Figure 7 illustrates to which extent individual elements influence the RSC score for the applied wavelength and laser irradiance. It plots the quotient |1 − RSC|/(1 + RSC) for each element that is expected to become smaller the smaller the deviation between the measured abundance and the certified abundance is. The quotient for the nonmetallic elements from the 775‐nm measurement (green triangles) shows a clear decreasing trend with increasing laser irradiance, which is not observed for the two shorter wavelengths. With the exception of the first measurement point, the metallic elements (see e.g., Mn at 775 nm, green triangles) exhibit a similar quotient to the one calculated for the two shorter wavelengths. This means the accuracy of the measured abundance for the IR radiation near the ablation threshold is particularly affected by the nonmetallic elements. At laser irradiances of about 3.1 TW/cm^2^ and higher, the quotients converge and are at a comparable level, as summarised visually in simplified Figure 6.

## 4 Conclusions

In comparison with laser systems with longer pulses, the introduction of femtosecond laser ablation ion sources allowed for the improvement of various figures of merit of nowadays laser‐based mass spectrometric systems, including but not limited to the minimisation of fractionation effects due to for example, laser–plasma interaction, improvement of the overall stoichiometry and clean material ablation. But still, femtosecond laser systems are more expensive than picosecond or nanosecond laser systems, which make femtosecond laser systems less attractive. Moreover, the generation of shorter wavelengths results in additional hardware and maintenance costs. In this study, the performance of the fundamental (775 nm), the second (358 nm) and third harmonics (258 nm) of a femtosecond laser system concerning the quantification of the chemical composition of metallic solids using LIMS is investigated. The systematic study consists of the application of wide ranges of applied pulse energies for each of the three wavelengths and the recording of 100,000 spectra at each instrument condition. The NIST alloy sample SRM 664 applied for microanalysis studies was used in this campaign. After filtering out not sufficiently resolved spectra and spectra showing deviating chemical composition due to element inhomogeneities of the SRM, laser irradiance ranges were identified where a comparable performance concerning element quantification can be reached, here about 3.1 TW cm^−2^ and higher. Only at the lower end of laser irradiances for each specific wavelength, significant deviations were observed. Overall, lower irradiances for shorter wavelengths needed to be applied to reach a comparable level of performance for quantification.

This study demonstrates that at least for a metallic sample, no need for the application of UV radiation is required, making the laser setup simpler, easier to maintain and at the same time cheaper. Nevertheless, shorter wavelengths (e.g., 358 nm) might be of high advantage in case materials transparent at IR wavelengths need to be investigated chemically, such as quartz‐based samples in geology.

## Author Contributions

**Valentine Grimaudo:** conceptualization, investigation, writing – original draft, methodology, validation, visualization, writing – review and editing. **Andreas Riedo:** investigation, writing – original draft, visualization, writing – review and editing, validation. **Marek Tulej:** investigation, validation, writing – review and editing, supervision. **Peter Wurz:** funding acquisition, validation, writing – review and editing, project administration, supervision, resources.

## Supporting information




**Figure S1:** SEM analysis of laser ablation craters formed on a Cu foil with 100 laser shots. (a) presents a crater formed by 0.6 μJ/shot at 358 nm, and (b) a crater formed by 2 μJ/shot at 775 nm. The inset in (a) shows the morphology of a single laser shot crater for 258 nm with 0.6 μJ/pulse.
**Figure S2:** Laser ablation crater areas formed on a Cu‐foil sample using the wavelengths 258, 387 and 775 nm at increasing pulse energies.
