# Supplementary material for: Wavelength Dependence of fs Laser Ablation Ionisation Mass Spectrometry: a Dedicated Study on NIST SRM 664
Source: Rapid Commun Mass Spectrom. 2025 Dec 16;40(5):e70012. doi: 10.1002/rcm.70012 (PMC12707028; doi:10.1002/rcm.70012)
Supplement: Supplementary file 1 — Figure S1: SEM analysis of laser ablation craters formed on a Cu foil with 100 laser shots. (a) presents a crater formed by 0.6 μJ/shot at 358 nm, and (b) a crater formed by 2 μJ/shot at 775 nm. The inset in (a) shows the morphology of a single laser shot crater for 258 nm with 0.6 μJ/pulse. Figure S2: Laser ablation crater areas formed on a Cu‐foil sample using the wavelengths 258, 387 and 775 nm at increasing pulse energies. [file RCM-40-e70012-s001.docx]

**Supporting Information**

Wavelength dependence of fs laser ablation ionisation mass spectrometry: a dedicated study on NIST SRM 664

Valentine Grimaudo^1^, Andreas Riedo^1*^, Marek Tulej^1^ and Peter Wurz^1^

^1^Physics Institute, Space Research and Planetary Sciences, University of Bern, Switzerland.


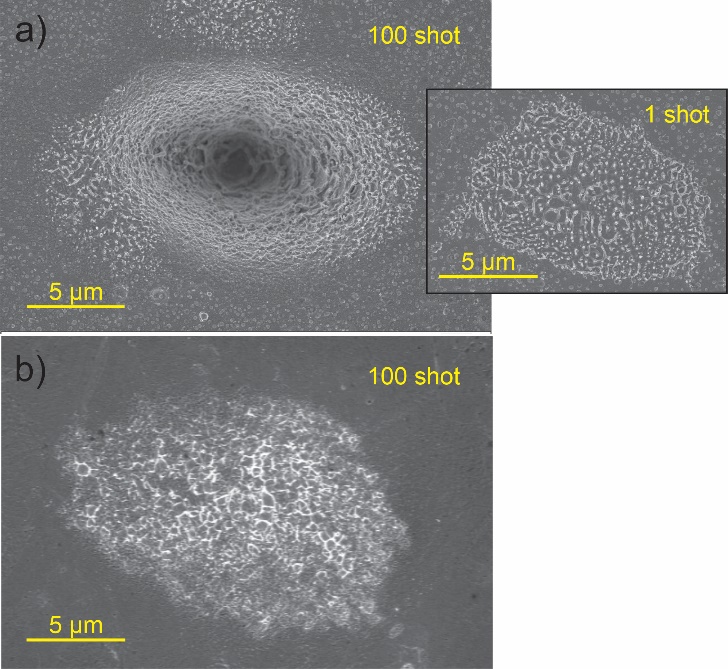


Figure S1 SEM analysis of laser ablation craters formed on a Cu foil with 100 laser shots. a) presents a crater formed by 0.6 µJ/shot at 358 nm, and b) a crater formed by 2 µJ/shot at 775 nm. The inset in a) shows the morphology of a single laser shot crater for 258 nm with 0.6 µJ/pulse.

In Figure S1 SEM images of two laser ablation craters formed with a) 387 nm and b) 775 nm wavelength after application of 100 laser pulses are shown, with 0.6 µJ/pulse and 2 µJ/pulse, respectively. The shorter wavelength removed for the same number of laser pulses, with lower pulse energy, a larger volume of the material and formed clearly a depression inside the substrate, whereas the IR radiation mainly modified the morphology of the surface creating melted structures and barely drilled a hole into the bulk. The efficiency of material removal is much higher for the shorter wavelengths. This implies that to obtain similar ablation volumes, a larger power density is required for IR radiation.

Figure S2 shows the growth of the crater area with increasing pulse energy, which is an exponential function, based on a study performed with the three different wavelengths on crater form on the Cu-foil sample. Because the Cu sample is expected to behave similarly to the Fe sample in terms of laser ablation, the derived exponential function for the crater size was used for the size of the crater areas on the NIST 664 sample to derive the applied laser irradiances. The spatial shape of the initial laser pulse from the fundamental beam at 775 nm is hardly modified by passing the harmonic generators (SHG or THG, respectively) given that the investigated crater areas fall all onto one line. Nevertheless, it should be noted that for the shorter wavelength it was possible to image the crater size from a single pulse, whereas for the 775 nm the crater area from 100 pulses had to be considered, as a single pulse did not result in any recognizable crater for the investigated pulse energy range (see Fig. S1). Because of accumulated defects that form in the surrounding area around the crater and contribute to its enlargement upon increasing number of applied laser pulses, it is expected to have a small discrepancy for the effective crater size for the 775 nm wavelength.


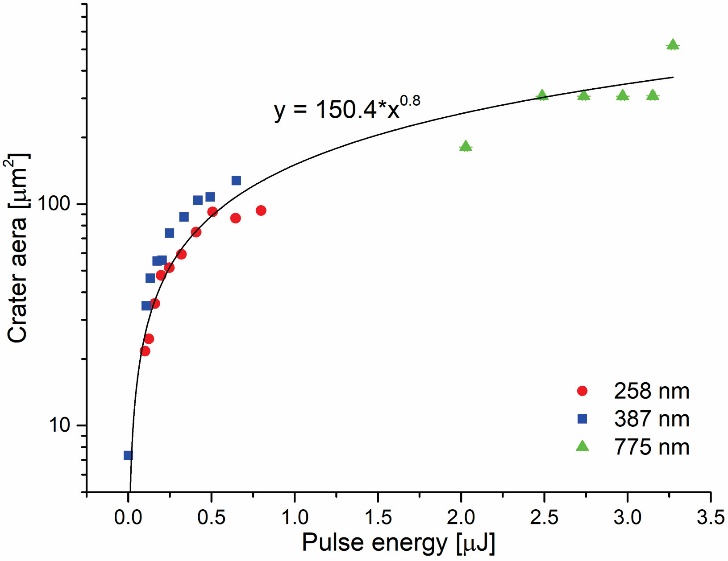


Figure S2 Laser ablation crater areas formed on a Cu-foil sample using the wavelengths 258 nm, 387 nm, and 775 nm at increasing pulse energies.
